# The Impact of Cosmetic and Plastic Surgery on Self-Esteem: A Systematic Review and Meta-analysis

**DOI:** 10.1093/asjof/ojag013

**Published:** 2026-01-29

**Authors:** Hugo Despert, Tarek Meniai, Cyril Bouland, Franck Dupuy, Guillaume Henry, Emmanuel Delay, Ali Mojallal

## Abstract

The global demand for cosmetic surgery continues to rise, driven largely by societal factors and personal motivations related to self-esteem and psychological well-being. Although previous reviews have examined general psychological outcomes, specific impacts on self-esteem remain underexplored. This systematic review and meta-analysis aimed to synthesize current evidence regarding the effects of nonreconstructive cosmetic surgery on self-esteem in adult patients. A systematic literature search was conducted across multiple databases, including MEDLINE, Embase, Cochrane Library, and others, from January 2005 to March 2025. Studies evaluating self-esteem before and after cosmetic procedures using validated scales were included. The methodological quality was assessed using the Effective Public Health Practice Project criteria. Data synthesis employed a random-effects meta-analyses using Hedges' *g* to estimate effect sizes. Twenty-five studies (*n* = 1502 patients) met inclusion criteria. The overall meta-analysis indicated a statistically significant improvement in self-esteem after cosmetic surgery (Hedges' *g* = 0.43, 95% CI, 0.13-0.73; *P* = .003). Subgroup analyses demonstrated a significant positive impact particularly in breast surgeries (*g* = 0.59; *P* = .006), whereas facial surgeries showed smaller, nonsignificant changes (*g* = 0.23; *P* = .268). Cosmetic surgery is associated with a small to moderate but significant improvement in self-esteem, particularly after breast procedures.

**Level of Evidence: 2 (Therapeutic)**  
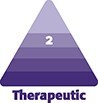

The global demand for cosmetic and plastic surgery has increased significantly in recent decades, driven by a growing societal preoccupation with physical appearance, continuous innovation in surgical techniques, and increased accessibility through social media and digital platforms. Although the motivations for undergoing aesthetic procedures are diverse, the pursuit of improved confidence, self-esteem, and well-being consistently emerges as a central factor in patient decision making.^1,2^

Self-esteem, understood as an individual's subjective assessment of self-worth, is a critical determinant of psychological well-being. It is closely related to mental health, interpersonal relationships, and overall quality of life.^3^ Low self-esteem has been associated with increased vulnerability to anxiety, depression, and body dysmorphic concerns.^4,5^

However, the relationship between cosmetic surgery and self-esteem remains nuanced and subject to ongoing debate. Individuals with low baseline self-esteem may be more likely to seek aesthetic interventions in response to perceived body dissatisfaction or self-image concern.^6,7^ On the other hand, although several studies have reported short-term gains in self-esteem following cosmetic procedures, the persistence and clinical significance of these effects over time are less well established.^8,9^ Understanding these dynamics is essential for refining patient selection, providing effective psychological support, and setting realistic expectations for surgical outcomes.

Given the continued increase in global demand for cosmetic procedures, a robust and up-to-date synthesis of the evidence on self-esteem outcomes is both timely and necessary.

To date, most systematic reviews and meta-analyses in this area have focused primarily on general psychological outcomes such as quality of life, body image, and depressive symptoms, often without isolating self-esteem as a specific outcome of interest.^8-10^ As a result, the specific impact of cosmetic surgery on self-esteem remains underexplored in the overall literature.

The present systematic review and meta-analysis aims to address this gap by evaluating the impact of nonreconstructive cosmetic surgery on self-esteem in adult patients. By synthesizing findings across different surgical procedures and populations, this review aims to elucidate the nature and magnitude of changes in self-esteem following aesthetic procedures and to provide clinicians with evidence-based guidance.

## METHODS

### Study Design and Registration

This study is a systematic review and meta-analysis of studies investigating self-esteem changes before and after plastic and cosmetic surgical procedures. The review was conducted in accordance with the Preferred Reporting Items for Systematic Reviews and Meta-Analyses (PRISMA) guidelines.^11^ The protocol was prospectively registered on the PROSPERO database (registration number: CRD420251017857).

### Articles Selection

A comprehensive literature search was performed in March 2025. It integrated the following databases: MEDLINE (through PubMed), Embase, Cochrane Library, Google Scholar, Web of Science, and Science Direct. Only studies published between January 2005 and March 2025 in English were considered. The search strategy used the following keywords and Medical Subject Heading (MeSH) terms: “self-esteem,” “cosmetic surgery,” “plastic surgery,” and excluded the terms “reconstructive surgery” or “reconstruction.” The detailed search string is shown in Appendix B.

Using the PICO framework, the population (P) was adult human subjects, interventions (I) were plastic or cosmetic surgery procedures, specifically excluding reconstructive surgeries (postcancer, trauma, or congenital malformations), comparisons (C) were primarily made using preintervention assessments within the same population, and outcomes (O) were self-esteem measurements by validated questionnaire such as the Rosenberg Self-Esteem Scale (RSES), the Coopersmith Self-Esteem Inventory (CSEI), or the extended self-esteem questionnaire (ESEQ).^12-15^ Exclusion criteria comprised studies without correlation data, qualitative designs, retrospective, or observational studies without pre–post comparisons, theses, conference abstracts, or case reports.

Initial screening of titles and abstracts was conducted by a single reviewer using the Rayyan platform (H.D.). When uncertainty regarding eligibility arose, a second reviewer was consulted, and decisions were reached through consensus (T.M.). In cases of persistent disagreement, a third reviewer adjudicated (C.B.). Full-text articles that passed the initial screen were independently reviewed by the same 2 reviewers. Reasons for exclusion at the full-text stage were systematically recorded.

Following the main database screening, backward and forward citation chasing was performed using Citationchaser, an automated tool developed to enhance comprehensiveness in systematic searches. References of included studies (backward search) and articles citing them (forward search) were screened. Any additional identified articles underwent the same selection process and eligibility assessment as the original dataset.

**Figure 1.**
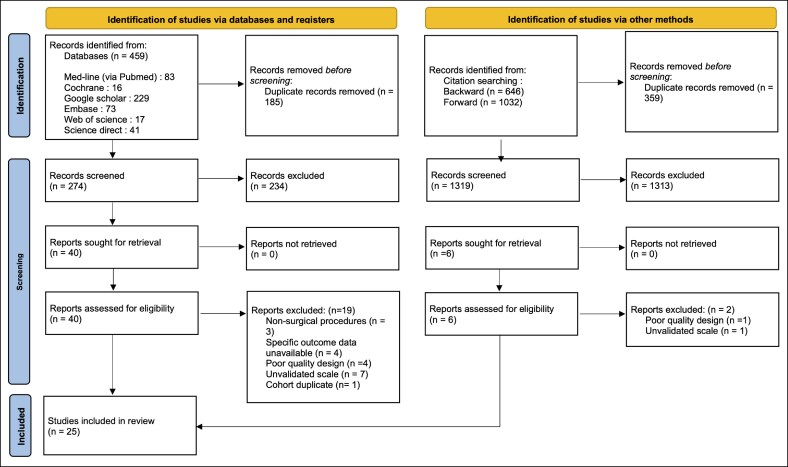
PRISMA flow chart of identified studies.

A detailed visual summary of the study selection process is presented in Figure 1 (PRISMA flow chart). The PRISMA checklist is shown in Appendix A.

### Data Extraction

Data extraction was conducted independently by 1 reviewer (H.D.). Extracted data included authors, year of publication, country, study design, sample size, participant demographics, surgical interventions, outcome measurement tools, time of outcome measurement, pre- and postintervention self-esteem scores.

### Data Synthesis and Statistical Analysis

Meta-analysis was planned if sufficient data were available, using the Meta-Essentials tools (Meta-Essentials: Workbooks for meta-analysis, Version 1.5). Mean differences (MDs) or standardized MDs (SMDs) and 95% CIs were calculated for continuous outcomes. Heterogeneity among studies was assessed using the *I*^2^ statistic. A random-effects model was employed for meta-analysis because of expected methodological heterogeneity. Subgroup analyses were planned according to type of surgical procedure. Hedges' *g* was used to calculate SMDs, because it corrects for small sample bias and allows comparison across studies using different scales. Effect sizes were interpreted using conventional thresholds, where values of ∼0.2 indicate small effects, 0.5 moderate effects, and ≥0.8 large effects.

### Data Standardization and Imputation

When studies reported self-esteem using modified or alternative versions of the RSES, scores were rescaled to the standard 10 to 40 range to ensure comparability across studies. In cases where standard deviations (SDs) were not provided, missing SDs were imputed using the mean of SDs from the remaining studies within the same subgroup. These procedures allowed the inclusion of all eligible data while maintaining methodological consistency.

### Risk of Bias Assessment

The risk of bias of the included studies was evaluated using the Quality Assessment Tool for Quantitative Studies developed by the Effective Public Health Practice Project (EPHPP). This framework appraises 6 key domains: selection bias, study design, control of confounders, blinding procedures, data collection instruments, and participant attrition. Based on these criteria, studies were assigned an overall quality rating of “strong” (no domains rated as weak), “moderate” (a single weak rating), or “weak” (2 or more domains rated as weak). Considering the heterogeneity in study designs among the included publications, the EPHPP was deemed the most suitable instrument for this review.

Quality assessment was carried out by 2 reviewers (H.D. and T.M.), with discrepancies resolved through discussion and consensus. Quality appraisal focused exclusively on components of the studies related to self-esteem outcomes. Studies rated as low quality for this purpose may nevertheless be considered robust with respect to other unrelated research objectives.

### Ethical Considerations

This review is based exclusively on extracted data from previously published literature; therefore, no ethical approval is required.

## RESULTS

The initial database search identified 459 articles, which was supplemented by citation chasing methods, which yielded an additional 1678 records. After removing duplicates (*n* = 544), a total of 1593 records were screened using titles and abstracts. Of these, 1537 articles were excluded as irrelevant, leaving 46 potentially eligible studies for detailed full-text review.

After comprehensive assessment, 19 studies were excluded. Citation chasing identified a further 6 potentially eligible studies, of which 2 were subsequently excluded (reasons for exclusion detailed in Figure 1). Finally, 25 studies that met all inclusion criteria were selected, with a total sample of 1502 patients. Overall, no studies were rated as low risk of bias, 21 as moderate, and 4 as high risk of bias according to the EPHPP criteria. Most moderate or high-risk ratings were attributable to lack of blinding and attrition.

Results are presented in 5 distinct categories: breast surgery, facial surgery, body contouring interventions, mixed procedures including heterogeneous surgical populations (different types of single aesthetic procedures across patients), and a final global overview.

### Breast Surgery

The category “breast surgery” comprised procedures such as breast reduction or breast augmentation and mastopexy. A detailed overview of the studies included in this subgroup is provided in Table 1. All studies using the RSES were included in the meta-analysis, with the exception of one in which it was not possible to standardize the self-esteem scores.

**Table 1.** Characteristics of Studies Evaluating Self-esteem After Breast Surgery

Author, year, reference	Country	Design	Type of surgery	*n* (M/W)	Age (years), mean ± SD (range)	Self-esteem scale	Date of post-op outcome assessments, months	Comparison test	Pre-op mean ± SD	Post-op mean ± SD	Δ (*P*-value)	Outcome	Quality bias assessment
O’Blenes et al, 2006^16^	Canada	PCWCG	Breast reduction	55 (0/55)	39.4 (21-61)	RSES	6	ANOVA	31	35	4 (.0001)	Improvement	Moderate
Neto et al, 2008^17^	Brazil	PRCT	Breast reduction	46 (0/46)	36.5^a^ (18-55)	RSES	6	ANOVA	31.1 ± 4.8^b^ Control: 30.9 ± 4.8^b^	35.1 ± 3.7^b^ Control: 31 ± 4.7^b^	4 (<.001) Control: 0.1 (>.999)	Improvement	Moderate
Kececi et al, 2015^18^	Türkiye	PCWCG	Breast reduction	78 (0/78)	41.6 ± 14.1 (18-60)	RSES	4	Paired *T*-test	28.7 ± 3.5	32.4 ± 3.1	3.7 (<.001)	Improvement	Moderate
Koraş Sözen and Karabulut, 2023^19^	Türkiye	PCWCG	Breast reduction	51 (0/51)	36.8 ± 10.6	RSES	6	Wilcoxon test	24.92 ± 4.23^c^	16.43 ± 3.16^c^	8.49 (.000)	Improvement	Moderate
Berenguel-Pérez and Cortés-Rodriguez, 2024^20^	Spain	PCWCG	Breast reduction	50 (0/50)	27.1 ± 6.4 (20-55)	RSES	1	Wilcoxon test	32 ± 3^d^	31 ± 5.3^d^	−1 (.94)	NSS	Moderate
Neto et al, 2012^21^ (similar population for breast hypertrophy already reported in previous study)	Brazil	PCWCG	Breast augmentation	40 (0/40)	NA	RSES	6	Paired *T*-test	32.35 ± 4.2	36.1 ± 3.71	3.75 (.001)	Improvement	Moderate
Klöppel et al, 2025^22^	Germany	PCWCG	Breast augmentation	50 (0/50)	30.8 ± 7.4 (20-44)	RSES	6	Paired *T*-test	29.2 ± 5.32	32.72 ± 4.61	3.41 (.00)	Improvement	Moderate
Chaleh Chaleh et al, 2017^23^	Iran	PCWCG	Breast reduction (*n* = 66) Breast augmentation (*n* = 19) Breast lifting (*n* = 15)	100 (0/100)	34.4^a^	RSES	2	Paired *T*-test	28.77 ± 4.5^d^	27.96 ± 3.39^d^	−0.81 (.94)	NSS	Weak

ANOVA, analysis of variance; NA, nonavailable; NSS, not statistically significant; PCCG, prospective cohort with control group; PCWCG, prospective cohort without control group; Post-op, postoperative; PRCT, prospective randomized controlled trial; Pre-op, preoperative; RSES, Rosenberg Self-Esteem Scale; SD, standard deviation. ^a^Approximate age calculated from available data in original articles when exact mean ± SD was not provided. RSES scores standardized to 10-40 range: ^b^Original scale 1-4 converted to 10-40. ^c^Unable to standardize RSES scores because of insufficient information provided in the original publication. ^d^Original score 0-30 converted to 10-40.

In 1 case, self-esteem score SD was not reported.^16^ To include it, an imputed SD was calculated based on the mean of the SDs reported in the remaining studies within the same subgroup.

The meta-analysis included 7 studies with a total of 419 participants. The majority (285 patients; 68.0%) underwent breast reduction, whereas 134 patients (32.0%) underwent other procedures such as breast augmentation or mastopexy.

**Figure 2.**
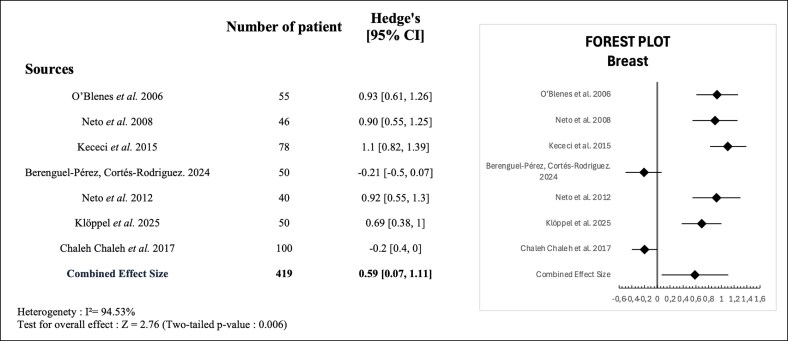
Forest plot of standardized mean differences (Hedges' *g*) in self-esteem before and after breast surgery across included studies.

It evaluated the changes in self-esteem before and after breast surgery using SMDs (Hedges' *g*). The aggregated effect size demonstrated a statistically significant improvement in self-esteem after surgery, with a pooled Hedges' *g* of 0.59 (95% CI, 0.07-1.11; *Z* = 2.76; *P* = .006). There was substantial heterogeneity between studies (*I*^2^ = 94.53%). These results are visually presented in the forest plot (Figure 2), illustrating both individual study contributions and overall estimated effect.

### Facial Surgery

Based on a re-evaluation of the eligible studies included (Table 2), 9 publications reporting facial cosmetic procedures including rhinoplasty, blepharoplasty, otoplasty, and rhytidectomy (facelift) were included for meta-analysis.

**Table 2.** Characteristics of Studies Evaluating Self-Esteem After Facial Surgery

Author, year, reference	Country	Design	Type of surgery	*n* (M/W)	Age (years), mean ± SD (range)	Self-esteem scale	Date of post-op outcome assessments, months	Comparison test	Pre-op mean ± SD	Post-op mean ± SD	Δ (*P*-value)	Outcome	Quality bias assessment
Alves et al, 2005^24^	Brazil	PCWCG	Facelift	32 (0/32)	55.1 (46-68)	RSES	6	ANOVA	33.38 ± (3.14)^b^	37.87 ± (1.83)^b^	4.49 (.001)	Improvement	Moderate
Jacono et al, 2016^25^	USA	PCWCG	Facelift	50 (2/48)	58 (37-73)	RSES	6	Paired *T*-test	34.3^c^	34.6^c^	0.3 (.69)	NSS	Moderate
Mousavi et al, 2018^26^	Iran	PCWCG	Rhinoplasty	83 (18/65)	24.9 ± 5.8 (17-48)	RSES	6	Paired *T*-test	22.6 ± 4.66	24.01 ± 3.73	1.41 (.002)	Improvement	Moderate
Borujeni et al, 2020^27^	Iran	PCWCG	Rhinoplasty	100 (37/63)	28.6^a^	RSES	3	Paired *T*-test	1.58 ± 0.25^d^	1.70 ± 0.17^d^	0.12 ± 0.31 (>.001)	Improvement	Moderate
Hashemi et al, 2020^28^	Iran	PCWCG	Rhinoplasty	41 (16/25)	30.5 (20-41)^a^	Extended self-esteem questionnaire (ESEQ)	6	Paired *T*-test	20.67 ± 5.53	22.73 ± 3.44	2.06 ± 0.68 (.004)	Improvement	Moderate
Chowdhury et al, 2022^29^	India	PCWCG	Rhinoplasty	24 (9/15)	25.6	RSES	6	Independent *T*-test	25.04	28.66	3.62 (<.05)	Improvement	Weak
Papadopulos et al, 2021^30^	Germany	PCWCG	Rhinoplasty	34 (5/29)	29.8 (15-53)	RSES	6	Paired *T*-test	33.24 ± 4.87	33.59 ± 4.81	0.35 (.591)	NSS	Moderate
Yagci and Gunay Yagci, 2024^31^	Türkiye	PCWCG	Rhinoplasty	86 (32/54)	24.0 ± 0.6	RSES	3	Paired *T*-test	32.6 ± 0.5	31.9 ± 0.5	− 0.7 (.06)	NSS	Moderate
Sirin et al, 2019^32^	Türkiye	PCWCG	Otoplasty	20 (6/14)	24.1 ± 8.3 (18-49)	RSES	6	Wilcoxon test	1.13 (0.5-1.84)^d^ Median (Q1;Q3)	0.8 (0.5-1.38)^d^ Median (Q1;Q3)	0.33 (.168)	NSS	Weak
Bashizadeh et al, 2018^33^	Iran	PCWCG	Blepharoplasty	60 (6/54)	56 (39-73)^a^	RSES	3	Paired *T*-test	32.91 ± 4.44	32.61 ± 4.49	−0.30 (.312)	NSS	Moderate
Papadopulos et al, 2023^34^	Germany	PCWCG	Blepharoplasty	50 (8/42)	54.7 (25-79)	RSES	6	Paired *T*-test	34.9	35.3	0.4 (.467)	NSS	Moderate
Yin et al, 2016^35^	China	PCCG	Blepharoplasty, rhinoplasty	126 (0/126)	24 (18-30)^a^	RSES	6	ANOVA	22.60 ± 1.80	25.88 ± 3.64	3.28 (.001)	Improvement	Moderate

ANOVA, analysis of variance; ESEQ, extended self-esteem questionnaire; NSS, not statistically significant; PCCG, prospective cohort with control group; PCWCG, prospective cohort without control group; Post-op, postoperative; Pre-op, preoperative; RSES, Rosenberg Self-Esteem Scale; SD, standard deviation. ^a^Approximate age calculated from available data in original articles when exact mean ± SD was not provided. RSES scores standardized to 10-40 range: ^b^Original reversed score (30-0) converted to 10-40. ^c^Original score 0-30 converted to 10-40. ^d^Unable to standardize RSES scores because of insufficient information provided in the original publication.

Studies that used non-RSES tools (eg, Hashemi et al using ESEQ), or reported self-esteem in formats incompatible with standardization (eg, Sirin et al reporting only medians and interquartile ranges, or Borujeni et al with unstandardized scores), were excluded from the quantitative synthesis despite qualitative relevance.^27,28,32^ This ensured consistency in calculating effect sizes and comparability between studies.

In 3 studies, SDs were not directly reported but were reliably inferred based on the mean of the reported SDs in the remaining studies within the same subgroup, allowing their inclusion.^25,29,34^

**Figure 3.**
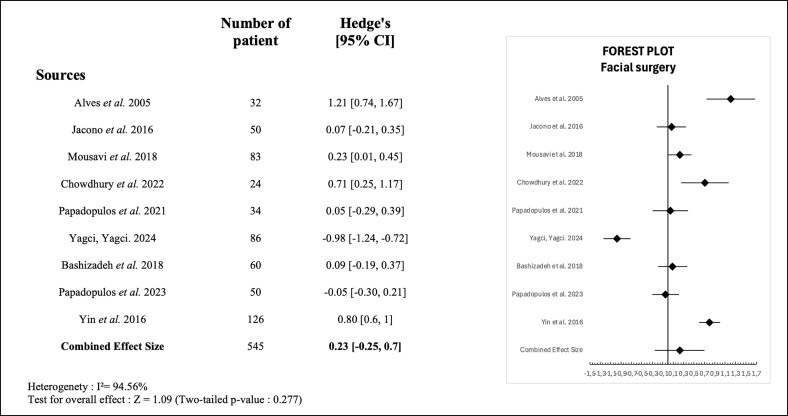
Forest plot of standardized mean differences (Hedges' *g*) in self-esteem before and after facial surgery across included studies.

The random-effects meta-analysis, which included 545 patients from 9 studies, yielded a pooled effect size (Hedges' *g*) of 0.23 (95% CI, −0.25 to 0.7). It indicates a small, nonsignificant improvement in self-esteem following facial cosmetic surgery (*Z* = 1.09, *P* = .277). Substantial heterogeneity was observed among the included studies (*I*^2^ = 94.56%). These results are shown in Figure 3.

### Body Contouring Surgery

Two studies evaluating the contribution of liposuction to self-esteem were identified and included in this review (Table 3). Both studies used the RSES and a prospective cohort design without control groups. Despite similarities in methodology and follow-up time, the 2 studies involved different patient populations and surgical indications, with insufficiently comparable data formats to allow quantitative synthesis; therefore, no meta-analysis was performed for this subgroup.

**Table 3.** Characteristics of Studies Evaluating Self-Esteem After Body Contouring Surgery

Author, year, reference	Country	Design	Type of surgery	*n* (M/W)	Age (years), mean ± SD (range)	Self-esteem scale	Date of post-op outcome assessments, months	Comparison test	Pre-op mean ± SD	Post-op mean ± SD	Δ (*P*-value)	Outcome	Quality bias assessment
Papadopulos et al, 2019^36^	Germany	PCWCG	Aesthetic liposuction	38 (6/32)	37.82 ± 11.85 (19-64)	RSES	6	Paired *T*-test	33.47 ± 5.10	33.34 ± 5.58	−0.13 (.88)	NSS	Moderate
Klöppel et al, 2024^37^	Germany	PCWCG	Liposuction for lipoedema	30 (0/30)	32.6 years (21-60)	RSES	6	Paired *T*-test	29.93 ± 6.35	33.33 ± 5.26	3.4 (.001)	Improvement	Moderate

NSS, not statistically significant; PCWCG, prospective cohort without control group; Post-op, postoperative; Pre-op, preoperative; RSES, Rosenberg Self-Esteem Scale; SD, standard deviation.

Papadopulos et al investigated the impact of aesthetic liposuction in a mixed gender cohort (*n* = 38) and reported no statistically significant change in self-esteem between baseline and follow-up (MD = −0.13, *P* = .88).^36^ Conversely, Klöppel et al evaluated the effect of liposuction in an all-female cohort of lipoedema patients (*n* = 30) and demonstrated a significant increase in self-esteem postoperatively (MD =+3.4, *P* = .001).^37^

### Mixed Procedures Study

Three studies reporting self-esteem outcomes after different types of aesthetic surgery, without stratification by procedure type, were included in a separate analysis (Table 4). Fernandes et al assessed changes in self-esteem in a cohort of 52 patients who benefited from mammoplasty, liposuction, or abdominoplasty.^39^ The authors observed a significant improvement in self-esteem 3 months after surgery (pre-op: 29.87 ± 2.10 vs post-op: 34.92 ± 1.84; *P* = .001) using the RSES.

**Table 4.** Characteristics of Studies Evaluating Self Esteem After Multiple Type of Surgery

Author, year, reference	Country	Design	Type of surgery	*n* (M/W)	Age (years), mean ± SD (range)	Self-esteem scale	Date of post-op outcome assessments, months	Comparison test	Pre-op mean ± SD	Post-op mean ± SD	Δ (*P*-value)	Outcome	Quality bias assessment
Akhlaghi et al 2015^38^	Iran	PCWCG	NA	46 (NA)	NA	CSEI	4	ANOVA	17.39 ± 3.89	18.82 ± 4.02	1.43 (.018)	Improvement	Weak
Fernandes Moreira Filho et al 2023^39^	Brazil	PCWCG	Mammoplasty Liposuction Abdominoplasty	52 (4/48)	37 ± 11	RSES	3	Paired *T*-test	29.87 ± 2.10	34.92 ± 1.84	5.05 (.001)	Improvement	Moderate
von Soest et al 2009^40^	Norway	PCCG	Breast reduction (*n* = 52) Breast augmentation (*n* = 55) Breast lifting (*n* = 20) Liposuction (*n* = 26) Abdominoplasty (*n* = 22) Blepharoplasty (*n* = 14) Scar correction (*n* = 1)	160 (5/155)	37.1	RSES	6	Paired *T*-test	30.9 ± 4.9^a^	31.6 ± 4.7^a^	0.7 (.02)	Improvement	Moderate

ANOVA, analysis of variance; CSEI, Coopersmith's Self-Esteem Inventory; NA, nonavailable; PCCG, prospective cohort with control group; PCWCG, prospective cohort without control group; Post-op, postoperative; Pre-op, preoperative; RSES, Rosenberg Self-Esteem Scale; SD, standard deviation. RSES scores standardized to 10-40 range: ^a^Original scale 1-4 converted to 10-40.

von Soest et al assessed 160 participants who underwent a wide range of aesthetic procedures including breast surgery, body contouring, and facial surgery.^40^ A modest but statistically significant increase in self-esteem was reported 6 months after surgery (30.9 ± 4.9 to 31.6 ± 4.7; *P* = .02). Akhlaghi et al found a small but significant increase in self-esteem scores 4 months after surgery in 46 patients (17.39 ± 3.89 to 18.82 ± 4.02; *P* = .018) using the CSEI.^38^

These studies could not be usefully included in subgroup meta-analyses, because of the lack of disaggregated data (procedure type and variation in outcome measures). However, they provide relevant observational evidence to support the potential psychological benefits of cosmetic surgery across different populations and types of procedures.

### Overall Analysis of Self-Esteem Outcomes Across All Procedures

To provide an overview of the relationship between cosmetic or plastic surgery procedures and self-esteem, we conducted a global meta-analysis including all the studies using the RSES and reporting pre- and postintervention data in a suitable format for effect size calculation. A total of 20 studies with a combined sample size of 1244 patients were included.

**Figure 4.**
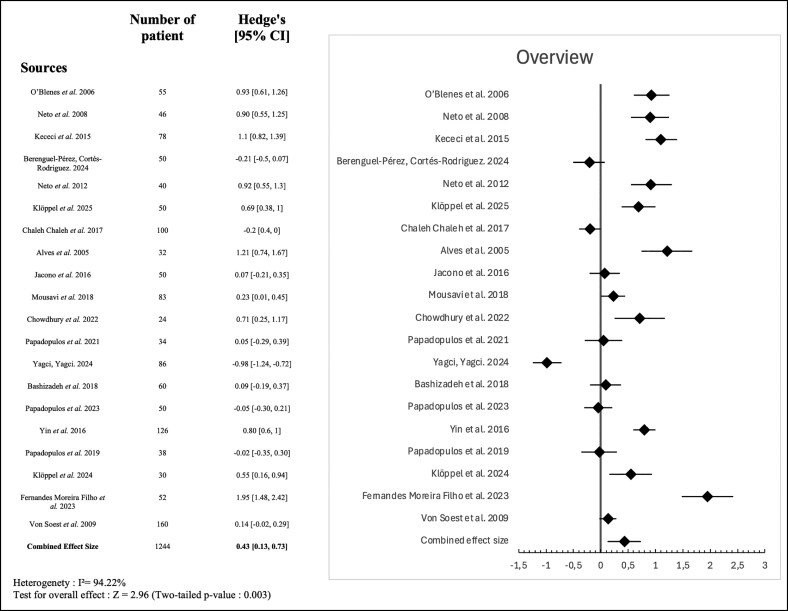
Forest plot of standardized mean differences (Hedges' *g*) in self-esteem before and after plastic and cosmetic surgery across included studies.

The analysis yielded a pooled Hedges' *g* of 0.43 (95% CI, 0.13-0.73; *P* = .003), suggesting a statistically significant improvement in self-esteem following surgery. This effect size reflects a small-to-moderate positive psychological benefit. There was considerable heterogeneity among the included studies (*I*^2^ = 94.22%). The forest plot illustrating these results is shown in Figure 4.

## DISCUSSION

This systematic review and meta-analysis explored the impact of cosmetic surgical procedures on self-esteem, an essential determinant of psychological well-being with broad implications for mental health, social interactions, and overall quality of life. Our findings demonstrated a statistically significant improvement in self-esteem following aesthetic interventions, aligning with conclusions drawn in previous research.^8-10^ The subgroup categorization, breast surgery, facial procedures, body contouring, and mixed interventions, allowed the exploration of potential variations according to anatomical regions. Specifically, breast surgery showed a clear statistically significant improvement, underscoring the critical role breast appearance plays in female self-concept.^41,42^ Conversely, outcomes in the facial surgery subgroup were less consistent, possibly reflecting the complexity of the relationship between facial appearance and self-esteem.^43,44^

The multidimensional and dynamic nature of self-esteem further complicates interpretation. Although many studies indicated positive changes postoperatively, others reported no improvement or even a decline in self-esteem, suggesting that the psychological benefits of cosmetic surgery are neither uniform nor guaranteed. Negative or negligible changes may be linked to unmet patient expectations, complications, or psychological phenomena, such as cognitive dissonance during early postoperative recovery.^45^ Furthermore, follow-up duration emerged as a crucial factor in interpreting the durability of self-esteem changes. Most included studies reported relatively short follow-up periods (3-6 months), which may be insufficient to capture the full psychosocial adaptation trajectory or the stability of postoperative outcomes. Existing long-term data provide important context. For example, a 5-year analysis by von Soest et al, although excluded from our quantitative synthesis to avoid duplication, demonstrated that improvements in self-esteem may be sustained or even amplified over time, suggesting durable psychological benefits in a subset of patients.^46^ Conversely, an 11-year follow-up study by the same authors reported poorer long-term mental health outcomes, indicating that the benefits of cosmetic surgery may diminish in the very long term or become influenced by broader life circumstances.^47^ By highlighting these divergent trajectories, long-term studies underscore the need for future research to distinguish between short-lived improvements and truly sustained psychosocial change and to identify which patient or procedural factors predict long-term durability.

Baseline psychological profiles of patients pursuing cosmetic interventions typically indicate a lower self-esteem compared with the general population, suggesting that the initial psychological status could influence the magnitude of postoperative improvement.^48,49^ Patients with lower baseline self-esteem might derive more pronounced subjective benefits from cosmetic procedures. Additionally, self-esteem is closely interconnected with other psychosocial outcomes such as body image, social functioning, and overall quality of life.^50,51^ Enhancements in self-esteem may therefore translate into improved interpersonal relationships, professional confidence, resilience against anxiety and depressive disorders, and even reduced healthcare use, an important point for healthcare policymakers and insurers.^51-53^

This meta-analysis deliberately focused on cosmetic surgical procedures performed with aesthetic intent, excluding reconstructive surgeries (eg, posttraumatic, postoncologic, or congenital corrections) because of their distinct psychosocial motivations and outcome expectations. This choice maintained conceptual homogeneity and ensured a more direct attribution of self-esteem changes to aesthetic motivations rather than would be functional restoration or medical necessity.

This study presents several key strengths. First, it clearly addresses the specific impact of cosmetic surgery on self-esteem. That question has been underexplored compared with broader psychosocial outcomes. Second, the comprehensive search strategy, including backward and forward citation chasing, ensured a rigorous identification of the relevant literature, thereby reducing publication bias. Moreover, methodological robustness was enhanced through the exclusive selection of studies using validated instruments, such as the RSES. It facilitates reliable comparisons and aggregation of data. The categorization into subgroups (breast, facial, body contouring, and mixed surgeries) allowed for more granular results interpretation, capturing potential anatomical differences in psychological responses. Finally, including studies that were initially excluded into a comprehensive global meta-analysis preserved valuable data, contributing to a more nuanced understanding of the overall psychosocial benefit. Collectively, these methodological approaches strengthened the validity of our findings and enhanced their relevance for clinical decision making, patient counseling, and policy formulation.

Our study has several methodological and conceptual limitations to be considered. Most included studies used nonrandomized designs, often relying on preexisting or nonequivalent comparison groups. This raises critical concerns about the appropriateness of control group selection and the adequate management of potential confounders. Nevertheless, many studies did not explicitly address these issues in their analyses. In addition, high rates of participant withdrawal and attrition were observed, potentially affecting the generalizability and external validity of our findings. An almost universal lack of information on blinding of participants and outcome assessors made the studies particularly susceptible to performance bias.

In addition, differences in study quality and methodological rigor across included studies may have influenced the magnitude and stability of the observed effect sizes. Studies with weaker designs or insufficient control of confounders may either overestimate or underestimate postoperative changes in self-esteem. This variability further underscores the need for cautious interpretation of the pooled results.

The decision to begin the literature search in 2005 was intentional. Earlier studies often involved outdated surgical techniques or nonvalidated psychological instruments, which would have reduced methodological comparability across included studies. Restricting the time frame to the modern era of aesthetic surgery also aligns with previous systematic reviews in this field, facilitating consistency with the existing literature. However, this choice may have excluded older studies that could offer historical insight into self-esteem outcomes. This temporal restriction should therefore be considered a potential limitation, as earlier evidence might have influenced the magnitude or variability of the observed effects.

Beyond these quality issues, the multifaceted motivations behind seeking cosmetic interventions further complicate interpretation. Some surgeries classified as cosmetic may have functional motivations, such as reduction mammaplasty or lipoedema treatment. These procedures commonly address significant functional impairments, including chronic pain, restricted physical mobility, or discomfort associated with daily activities. Consequently, the observed enhancement in self-esteem following these surgeries might be amplified by simultaneous improvements in overall functionality and quality of life. This dual effect underscores the multidimensional nature of self-esteem; improvement is not solely dependent on aesthetic appearance but is also significantly influenced by the relief of functional limitations. Importantly, distinguishing purely aesthetic procedures from functionally overlapping ones may help clarify heterogeneous findings and better anticipate psychological responses. Future research should consider stratifying interventions accordingly and leveraging emerging tools such as artificial intelligence assisted preoperative counseling and PROMs integration to capture these nuanced trajectories more systematically. Moreover, although aesthetic and functional motivations frequently overlap in plastic surgery, differences in primary indications may influence postoperative psychosocial trajectories. These varying motivations could partly contribute to the inconsistent findings observed across studies.

Significant heterogeneity was evident in our meta-analysis (*I*^2^ > 90%), reflecting variability in patient demographics, baseline psychological characteristics, surgical techniques, follow-up durations, and cultural contexts. This level of variability necessitates a cautious and nuanced interpretation of the findings. Indeed, several included studies reported no significant or even negative changes in self-esteem postoperatively, underscoring the influence of factors such as unmet expectations, postoperative complications, cognitive dissonance during early recovery, and the overall complexity of psychological outcomes.

Another important limitation relates to the predominance of short follow-up durations. Most studies assessed outcomes at 3 to 6 months postsurgery, which is likely insufficient to capture the full trajectory of psychosocial adaptation. Long-term data, such as the 5-year follow-up by von Soest et al, suggest that improvements in self-esteem may not only persist but, in some cases, intensify over time, highlighting a crucial gap and the need for extended longitudinal research.^46^

Additionally, imputations performed to estimate missing SDs, although methodologically necessary to retain studies in the quantitative synthesis, introduced a degree of uncertainty that may affect the precision of calculated effect sizes. Sensitivity analyses were considered to test the robustness of the meta-analytic findings. However, because of the small number of studies available within each subgroup, the substantial methodological heterogeneity between studies, and the limited availability of disaggregated data, formal sensitivity analyses (such as leave-one-out or exclusion based on study quality) could not be conducted without significantly reducing statistical power or compromising validity.

Several studies were excluded from quantitative analysis because of methodological shortcomings or incomplete reporting; nonetheless, their qualitative findings consistently supported the beneficial effects of cosmetic surgery on self-esteem and body image. This convergence reinforces the robustness of our conclusions but also emphasizes the urgent need for consistent, rigorous, and standardized reporting practices.

Ultimately, despite the encouraging evidence, the absence of homogeneous, prospective studies with large sample sizes and long-term follow-up remains a critical limitation. Our ongoing multicenter prospective study was specifically designed to address these shortcomings and will provide robust longitudinal data to better characterize patient outcomes.

Furthermore, the generation of rigorous scientific evidence is paramount, not only to justify the continued practice of aesthetic surgery, but also to highlight its significant therapeutic role. Such evidence has critical societal implications, underpinning policies that exempt aesthetic procedures from taxation in many countries because of their recognized therapeutic value. Ultimately, strengthening the therapeutic legitimacy of aesthetic surgery through sound scientific data is essential for its continued acceptance, practice, and accessibility worldwide.

## CONCLUSIONS

This systematic review and meta-analysis demonstrate that cosmetic surgery is associated with a statistically significant self-esteem improvement (Hedges' *g* = 0.43; 95% CI, 0.13-0.73), suggesting small-to-moderate psychological benefits. However, this effect must be interpreted with caution because of substantial heterogeneity across studies, likely reflecting differences in surgical procedures, patient populations, follow-up durations, and baseline psychological profiles.

## Supplemental Material

This article contains supplemental material located online at https://doi.org/10.1093/asjof/ojag013.

## Supplementary Material

ojag013_Supplementary_Data
