# Supplementary material for: The Impact of Cosmetic and Plastic Surgery on Self-Esteem: A Systematic Review and Meta-analysis
Source: Aesthet Surg J Open Forum. 2026 Jan 29;8:ojag013. doi: 10.1093/asjof/ojag013 (PMC12934344; doi:10.1093/asjof/ojag013)
Supplement: ojag013_Supplementary_Data [file ojag013_supplementary_data.zip › Appendix B.docx]

| Databases | Search terms | Filters | Search date | Results |
| --- | --- | --- | --- | --- |
| Medline (via pubmed) | (("Self-Esteem"[Title/Abstract] AND ("Cosmetic Surgery"[Title/Abstract] OR "Plastic Surgery"[Title/Abstract] OR "Aesthetic Surgery"[Title/Abstract] )) NOT ("Reconstructive Surgery"[Title/Abstract] OR "Reconstruction"[Title/Abstract] )) AND ((y_10[Filter]) AND (humans[Filter]) AND (english[Filter]) AND (alladult[Filter])) | Publication date : 2005-2025  LANGUAGE : ENGLISH  Adult | 13 March 2025 | 83 |
| Cochrane | ("Self-Esteem") AND ("Cosmetic Surgery" OR "Plastic Surgery" OR "Aesthetic Surgery") NOT ("Reconstructive Surgery" OR "Reconstruction") | Publication date : 2005-2025  LANGUAGE : ENGLISH | 13 March 2025 | 16 |
| Google scholar | allintitle: AND AND Cosmetic OR Surgery OR Plastic OR Surgery OR Aesthetic OR Surgery "Self esteem" -Reconstructive -Reconstruction | Publication date : 2005-2025  LANGUAGE : ENGLISH | 13 March 2025 | 229 |
| Embase | ('self esteem'/exp OR 'self esteem') AND 'plastic surgery' AND 'esthetic surgery' NOT 'reconstructive surgery' AND [2015-2025]/py | Publication date : 2005-2025  LANGUAGE : ENGLISH | 13 March 2025 | 73 |
| Web of science | ("Self-Esteem") AND ("Cosmetic Surgery" OR "Plastic Surgery" OR "Aesthetic Surgery") NOT ("Reconstructive Surgery" OR « Reconstruction")  Title/Abstract | Publication date : 2005-2025  LANGUAGE : ENGLISH | 13 March 2025 | 17 |
| Science direct | ("Self-Esteem") AND ("Cosmetic Surgery" OR "Plastic Surgery" OR "Aesthetic Surgery") NOT ("Reconstructive Surgery" OR "Reconstruction") | Publication date : 2005-2025  LANGUAGE : ENGLISH | 13 March 2025 | 41 |
| Citationchaser | Total :  Backward : 646  Forward : 1032 | Duplicate : 72 |  |  |
